# Strain engineering of electronic properties and anomalous valley hall conductivity of transition metal dichalcogenide nanoribbons

**DOI:** 10.1038/s41598-022-13398-5

**Published:** 2022-07-04

**Authors:** Farzaneh Shayeganfar

**Affiliations:** Department of Physics and Energy Engineering, Amirkabir University of Technology, Tehran, Iran

**Keywords:** Materials science, Physics

## Abstract

Strain engineering is a powerful technique for tuning electronic properties and valley degree of freedom in honeycomb structure of two-dimensional crystals. Carriers in + k and − k (opposite Berry curvature) in transition metal dichalcogenide (TMD) with broken inversion symmetry act as effective magnetic fields, where this polarized valleys are suitable for encoding information. In this work, we study the strained TMD nanoribbons by Slater-Koster tight-binding model, which acquires electronic bands in whole Brillouin zone. From this, we derive a generic profile of strain effect on the electronic band structure of TMD nanoribbons, which shows indirect band gap, and also exhibits a phase transition from semiconductor to metallic by applying uniaxial X-tensile and Y-arc type of strain. Midgap states in strained TMD nanoribbons are determined by calculation of localized density of electron states. Moreover, our findings of anomalous valley Hall conductivity reveal that the creation of pseudogauge fields using strained TMD nanoribbons affect the Dirac electrons, which generate the new quantized Landau level. Furthermore, we demonstrate in strained TMD nanoribbons that strain field can effectively tune both the magnitude and sign of valley Hall conductivity. Our work elucidates the valley Hall transport in strained TMDs due to pseudo-electric and pseudo-magnetic filed will be applicable as information carries for future electronics and valleytronics.

## Introduction

Valley Hall effect (VHE) in transition metal dichacogenides (TMDs) emerges due to inversion symmetry breaking. Lack of space inversion symmetry or time-reversal symmetry or both in materials systems leads to anomalous velocity of electrons and consequently create anomalous Hall current^1–5^. The electronic structure under external stimuli can change Berry curvature and evolve intrinsic anomalous Hall effect^6–11^.

To improve device performance, strain as useful tool changes the band gap, the effective mass and electrons mobility^12–14^. Moreover, vibrational modes by strain become hard and soft in two-dimensional (2D) materials, which is confirmed by micro Raman spectroscopy^15^. Compressive strained monolayer MoS_2_ at about 5% exhibits transition from a direct to an indirect band gap^16,17^. Meanwhile, the single photon emission of thin semiconductors is tuned by elastic strain engineering. For instance, rippled graphene under extreme (> 10%) strain creates short wavelength and periodic pseudogauge-fields due to large variation of carbon–carbon length and spatially oscillating strain field, which yield to new Lnadau quantization^18^.

Dopants and defects induced Midgap states, which play a key role in the electronic transport properties of 2D semiconducting TMDs as well as light absorption and emission of materials^19,20^. Several reports have been established to investigate the electronic structure and spatial configuration of doped TMDs by using scanning tunneling microscopy (STM)^21–25^.

Illumination of monolayer MoS_2_ transistors by polarized light causes to exciting electrons into a specific valley, where an anomalous Hall voltage is found and its sign is controlled by the helicity of the light^26^. However, in bilayer devices anomalous Hall effect is not observed, because of the restoration of crystal inversion symmetry^26^.

More recently, Iff et al.^27^ showed that localized quantum emitters in wrinkled WSe_2_ monolayers can emit a single photon, which is controlled by strain fields due to a hybrid 2D semiconductor-piezoelectric device. In general, the electronic properties of MoS_2_ is changed by strain for 15% compressive and 8% tensile strain, where semiconductor to metal transitions have been observed^28–32^.

Local extrema as inequivalent valleys in the k-space i.e. electron band structure of TMDs represents the electron valley degree of freedom, which can be as significant information carriers tunable via external fields^28,33–37^. To manipulate electron valley degree of freedom, a proper means can be VHE^28,38–40^. Similar to an ordinary Hall effect, in which by applying a uniform magnetic field in real space cause to drive a transverse charge current, a transverse valley current in the VHE in k-space is created by valley-contrasting Berry curvatures^18,41–49^, which traverse carriers in opposite direction from different valleys by the application of an external electric field. Therefore, 2D hexagonal materials with K and K′ valleys in the Brillouin zone exhibit VHE suitable for valleytronics^18^. Son et al.^50^ applied strain to the monolayer TMD, which induces the Berry curvature dipole, enabling the mechanical tuning of valley magnetization due to in-plane electric field. In other study by Xu et al.^51^ fully spin- and valley-polarized anomalous Hall conductivity have been obtained in the WS_2_/MnO heterostructure as a result of large valley splitting and time-reversal symmetry broken.

In this work, we carry out systemic electronic structure calculations based on tight-binding (TB) approach to investigate the electronic properties and valley Hall transport in both X-tensile- and Y-arc strained TMD nanoribbons. We find that (1) as the uniaxial strain increases, TMD nanoribbons exhibit a transition from semiconductor to metal, where valance valleys are shifted towards positive energies. Also, Midgap states in strained TMD nanoribbons are tuned with the strain field. (2) The sign and magnitude of anomalous valley Hall conductivity (AVHC) of TMD nanoribbons has been changed with strain. (3) In TMD nanoribbons under strain, psedomagnetic field affects the Berry curvature and creates exotic surface states, while the evolution of Berry curvature correlates with nonmonotonic change of AVHC. These results altogether suggest that strain field play as a powerful technique for tuning the quantum electronic states as well as Berry curvature and AVHC applicable in a wide range of quantum advanced materials.

## Methods and model description

**Figure 1:**
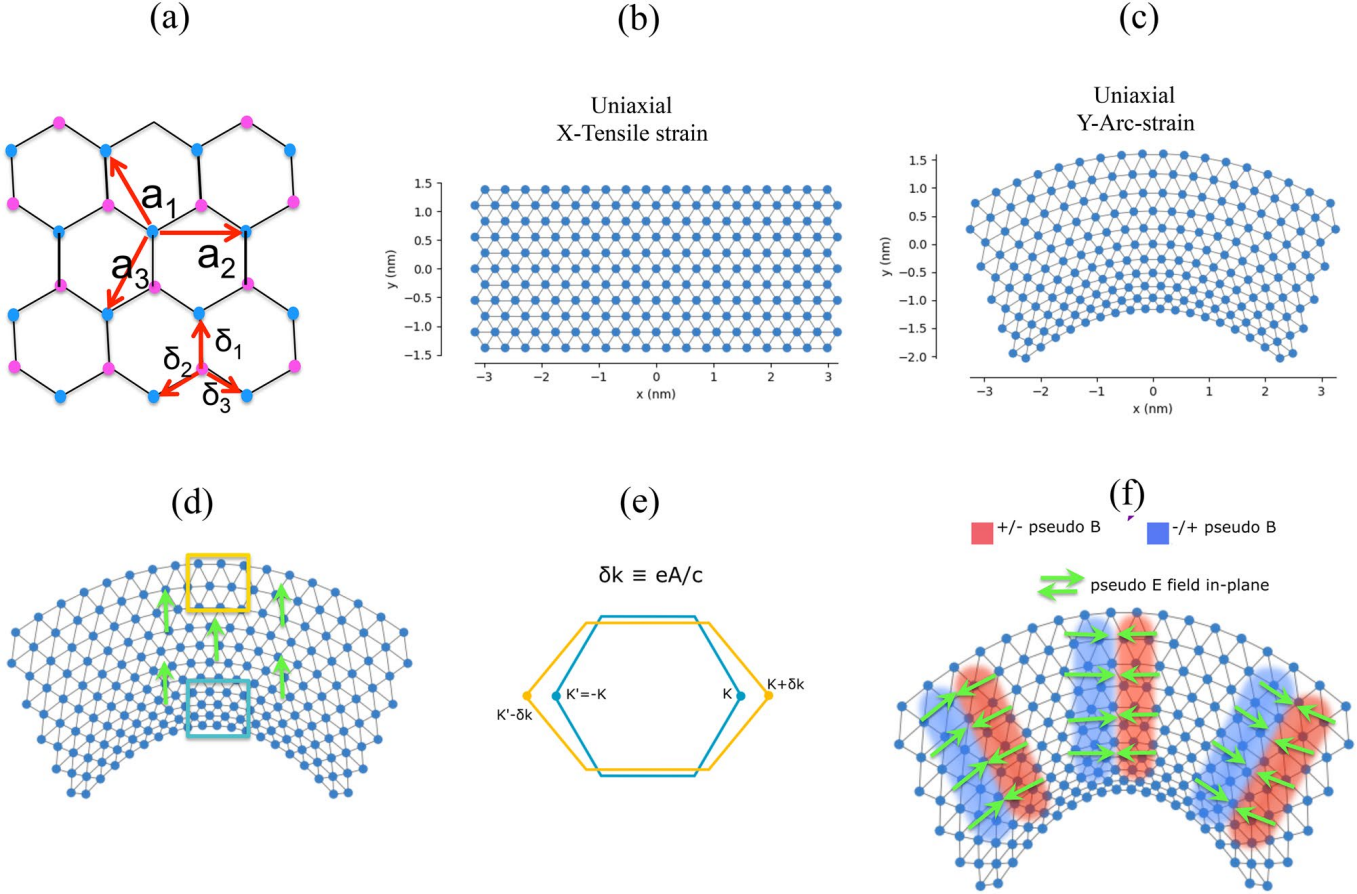
(**a**) Top view of unit cell of MX_2_, where δ_i_ are the nearest neighbor and a_i_ are the next nearest neighbors. Schematic of MX_2_ nanoribbons for (**b**) X-tensile strain and (**c**) Y-arc strain. Engineering pseudo-electric/magnetic fields at strained interfaces: (**d**) High density of atoms and electrons are created by strain in regions indicated by blue box and low density of atoms indicated by yellow box, which inhomogeneous charge distribution generates an electric field (green arrows). (**e**) Yellow hexagonal shows the stretching of bonds results in shift in Dirac cones at K and K′ points from their unstrained positions (blue hexagonal) in the reciprocal space. This momentum shift δk introduced a pseudovector potential term eA/c, which creates opposite sign pseudomagnetic fields at the two valleys. (**f**) The strain associated with pseudofields both electric fields (green arrows) and magnetic fields, where two blue/red regions indicate the ± ẑ direction of pseudospin up and down.

In this study, we model six strained TMD nanoribbons i.e., MoS_2_, MoSe_2_, MoTe_2_, WSe_2_, WSe_2_ and WTe_2_ using TB model. A finite-size TMD nanoribbons in one direction is constructed, then we have to passive or remove a few dangling bonds on the edge of the system. These are not desired and we can remove these dangling bonds in our TB model by setting lattice neighbors attribute. In the TB model, we used the minimum lattice neighbors method, which is required to remove any atoms, which have less than the specified minimum number of neighbors. We consider two types of strained structure as represented in Fig. 1, as named as uniaxial X-tensile and uniaxial Y-arc strain with several displacement such as, δ = 0, 0.02, 0.04, 0.06, 0.08, 0.1, 0.15, 0.2. Six monolayer TMDs have a direct band gap at the K and K′ points of the hexagonal Brillouin zone (BZ), which behave as a semiconductor.

Ab-initio calculations reveal two additional secondary extrema, that a local minimum of conduction band (CB) is located at Q point, while a local maximum of the valence band (VB) is located at the Γ point, midway between Γ and K point^52^. These features are not consistent with their optical properties and transport^53,54^. The massive Dirac Hamiltonian describe the low-energy K and K′ points of monolayer MoS2^45^.

TB Hamiltonian^55–57^ and k.p approximation^56,58^ has been developed as accurate approximations beyond the massive Dirac model, which take into account for diagonal quadratic terms in momentum and the presence of trigonal warping. In this section, we employ TB Hamiltonian for calculating the electronic band structure of strained TMDs.

### Tight binding model for strained TMDs

The Slater-Koster TB Hamiltonian in Ref.^59^ captured the electronic band structure of monolayer MoS_2_ in the whole BZ, including 11 bands of the d orbitals of the transition metal (Mo) and the p orbitals of the chalcogenide (S) atoms. It’s worth to note that the physics of monolayer MX_2_ around the band gap can be obtained by performing an unitary transformation in the subspace that transform the p orbitals of the bottom and top X layers into their combination of symmetric and antisymmetric with respect to the z-axis. For including the local spin–orbit interaction^60^, dominating of diagonal term L_z_S_z_ can be appear, which each spin sector can be dealt with separately^60^. Figure 1 represents a top view of the crystal lattice of MX_2_. The reduced Hilbert space can be considered using compact notation of Ref^59^:

1 $$\vec{\psi } = \left( {d_{{3z^{2} - r^{2} }} , d_{{x^{2} - y^{2} }} , d_{xy} , p_{x}^{S} , p_{y}^{S} , p_{z}^{A} } \right)$$

where the S and A superscripts refer to symmetric and antisymmetric of the p-orbitals combination of $p_{i}^{S} = 1/\sqrt 2 \left( {p_{i}^{t} + p_{i}^{b} } \right)$ and $p_{i}^{A} = 1/\sqrt 2 \left( {p_{i}^{t} - p_{i}^{b} } \right)$, here index *i* is spatial direction *i* = *x,y,z* and the superscripts of *t* stands for top and *b* for bottom X = S, Se plane. The TB Hamiltonian based upon base (Eq. 1) orbital hybridization, including the local spin–orbit coupling in the real space can be expressed:

2 $$H = \mathop \sum \limits_{i,\mu \nu } \epsilon_{\mu ,\nu } c_{i,\mu }^{\dag } c_{j,\nu } + \mathop \sum \limits_{ij,\mu \nu } \left[ {t_{ij,\mu \nu } c_{i,\mu }^{\dag } c_{j,\nu } + H.c.} \right]$$

where $c_{i,\mu }^{\dag } \left( {c_{i,\mu } } \right)$ creates (destroys) an electron in the atomic orbital of μ = 1,..,6 of Hilbert space of base (1) and in the unit cell i. A compact form of the TB Hamiltonian in the k-space is:

3 $$\begin{gathered} M = \left( {\begin{array}{*{20}c} {H_{MM} } & {H_{MX} } \\ {H_{{MX^{\dag } }} } & {H_{XX} } \\ \end{array} } \right) \hfill \\ H_{MM} = \epsilon_{M} + 2\mathop \sum \limits_{t = 1,2,3} t_{i}^{MM} {\text{cos}}\left( {k.a_{i} } \right) \hfill \\ H_{XX} = \epsilon_{X} + 2\mathop \sum \limits_{t = 1,2,3} t_{i}^{XX} {\text{cos}}\left( {k.a_{i} } \right) \hfill \\ H_{MX} = \mathop \sum \limits_{t = 1,2,3} t_{i}^{MX} e^{{ - ik,\delta_{i} }} \hfill \\ \end{gathered}$$

where the δ_i_ and a_i_ are the nearest and next nearest neighbor vectors are shown in Fig. 1. The hopping terms ${t}_{ij,\mu \nu }$ within a Slater-Koster approach have been considered^59–61^, which brought in Table 1.

**Table 1:** Slater-Koster TB parameters for monolayer MoX_2_.

	MoS_2_	MoSe_2_	WS_2_	WSe_2_
Crystal fields	− 0.050 − 1.511 − 6.886 − 7.503	− 0.250 − 1.488 − 4.931 − 7.327	− 0.650 − 2.279 − 3.864 − 6.759	− 1.250 − 2.321 − 5.629 − 3.559
M-X	3.689 − 1.241	3.728 − 1.222	7.911 − 1.220	5.803 − 1.081
M-M	− 0.895 0.252 0.228	− 0.823 0.192 0.215	− 1.328 0.121 0.442	− 1.129 0.094 0.317
X-X	1.225 − 0.467	1.256 − 0.205	1.178 − 0.273	1.530 − 0.123

Where M is Mo, W and X is S, Se. All terms are in units of eV and taken from Ref.^62^.

### Hamiltonian in strained lattice

The Slater-Koster TB approach for lattice deformations like strain is convenient^65^. In this approach, the effect of strain takes into account by considering of TB parameters of energy integral element of two-center energy dependent on the interatomic distances, which the correction to the local atomic potentials due to lattice deformation is neglected as a first approximation^63,64^. Here, we apply strain effect by varying the interatomic bond length in the simplest way. The modified hopping terms with strain at the linear order can be written as^65^:

4 $$t_{ij,\mu \nu } = t_{ij,\mu \nu } \left( {r_{ij}^{0} } \right)\left( {1 - {\Lambda }_{ij,\mu \nu } \frac{{\left| {r_{ij} - r_{ij}^{0} } \right|}}{{\left| {r_{ij}^{0} } \right|}}} \right)$$

where $$|{r}_{ij}^{0}|$$ is the distance in the absence of strain at the equilibrium positions between two atoms of (i,μ) and (j,ν), while |r_ij_| is the distance in the presence of strain. Here, $${\Lambda }_{ij,\mu \nu } = {\raise0.7ex\hbox{${ - dln t_{ij,\mu \nu } }$} \!\mathord{\left/ {\vphantom {{ - dln t_{ij,\mu \nu } } {dln\left( r \right)\left| {r = } \right|r_{ij}^{0} |}}}\right.\kern-\nulldelimiterspace} \!\lower0.7ex\hbox{${dln\left( r \right)\left| {r = } \right|r_{ij}^{0} |}$}}$$ is the local electron–phonon coupling^65^. In practice, $$|r_{{ij}}^{0} | = a$$ for in-plane *M-M* and *X-X* bonds and $$|r_{{ij}}^{0} | = \sqrt {\frac{7}{{12}}} a$$ for *M-X* bond have been applied^65^. In the absence of any theoretical and experimental estimation for the electron–phonon coupling, we use the Wills-Harrison argument^52^ as $${t}_{ij,\mu \nu }\left(r\right)\propto {|r|}^{-({l}_{\mu }+ {l}_{\nu }+1)}$$, where l_μ(ν)_ is the absolute value of the angular momentum of orbital μ(ν). Following this approach, $${\Lambda }_{ij,M - M} = 5, {\Lambda }_{ij,X - X} = 3$$ for *M-M* dd and for *X-X* pp hybridization and $${\Lambda }_{ij,X-M}=4$$ for *X-M pd* hybridization. The vector r_0_ as separation of two lattice site connected with electron hoping is transformed by application of strain into $$r \propto r_{0} + r_{0} . \nabla u$$^65^, where $$\nabla u=\varepsilon + \omega$$; ε is the strain tensor and ω is the rotation tensor. The strain tensor for 2D materials is a symmetric tensor as:

5 $$\varepsilon = \left( {\begin{array}{*{20}c} {\varepsilon_{xx} } & {\varepsilon_{xy} } \\ {\varepsilon_{xy} } & {\varepsilon_{yy} } \\ \end{array} } \right)$$

with components including *u*_*ii*_* i*s the in-plane and *u*_*ij*_ is the out-of-plane displacement as:

6 $$\varepsilon_{ij} = \frac{1}{2} \left( {\frac{{\partial u_{i} }}{{\partial r_{j} }} + \frac{{\partial u_{j} }}{{\partial r_{i} }}} \right) + \frac{1}{2} \frac{{\partial u_{z} }}{{\partial r_{i} }} \frac{{\partial u_{z} }}{{\partial r_{j} }}$$

where *u* = *(u*_*x*_, *u*_*y*_, *u*_*z*_*)* is the displacement vector and ***r* = **(*x,y*) is the position vector. To account the local rotation in the system, we use the *ω* as the anti-symmetric rotation tensor as defined: 2 *ω*_*xy*_ = − 2 *ω*_*yx*_ = $\left(\frac{{\partial u}_{y}}{\partial x}+ \frac{{\partial u}_{x}}{\partial y}\right)$*,* which *ω* for homogenous strain will be zero. It is worth to note that the transformation relation is ${\varvec{r}}= {{\varvec{r}}}_{0\boldsymbol{ }}. (1+{\varvec{\varepsilon}})$ for homogenous strain and ${\varvec{r}}=\boldsymbol{ }{{\varvec{r}}}_{0}\boldsymbol{ }.\boldsymbol{ }\boldsymbol{ }(1+\boldsymbol{ }\nabla {\varvec{u}})$ for inhomogeneous strain fields.

### Hall conductivity

Valence band (VB) and conduction band (CB) edges of monolayer MoS_2_ are located at the corners of K points of hexagonal plane^66^. The large separation of two inequivalent valleys in momentum space constitutes a binary index, which is robust against scattering by long wavelength phonons and deformation^45^. Therefore, coexistence of VHE in TMD monolayer has to be investigated similar to graphene. Broken inversion symmetry in TMD monolayers give rise to VHE with flowing carriers in different valleys by applying electric field. Calculation of quantum VHE of 2D electron gas indicates the quantized nature in unit of e^2^/ħ, which observed in graphene at room temperature^67^. In TMDs with time-reversal or broken inversion, pronounced Berry curvature can emerge VHE. Quantum Hall conductivity is arose due to anomalous velocity of electrons in the presence of an in-plane electric field, which is proportional to the Berry curvature in the transverse direction^7,68^, defined as:

7 $$\sigma_{xy}^{AHE} = \frac{{e^{2} }}{\hbar } \mathop \int \limits_{BZ}^{{}} \frac{{d\mathop{k}\limits^{\rightharpoonup} }}{{2\pi^{3} }} {\Omega }^{z} \left( {\mathop{k}\limits^{\rightharpoonup} } \right)$$

where z-component of Berry curvature $\Omega^{z} \left( {\mathop{k}\limits^{\rightharpoonup} } \right) = \mathop \sum \limits_{n} f_{n} \left( k \right)\Omega_{n}^{z} \left( k \right)$; is the sum of band resolved of Berry curvature or alternative expression defines as $\Omega_{n} \left( {\mathop{k}\limits^{\rightharpoonup} } \right) = \hat{z} . \nabla_{{\mathop{k}\limits^{\rightharpoonup} }} \times u_{n} \left( {\mathop{k}\limits^{\rightharpoonup} } \right)\left| { i\nabla_{{\mathop{k}\limits^{\rightharpoonup} }} } \right|u_{n} \left( {\mathop{k}\limits^{\rightharpoonup} } \right)$, is the Berry curvature in $\hat{z}$ direction and $f_{n} \left( {\mathop{k}\limits^{\rightharpoonup} } \right)$ is the Fermi–Dirac distribution function for the Bloch state *|nk* > . Furthermore, the Bloch state *|nk* > of Berry curvature i.e. ${\Omega }_{n}^{z} \left( k \right)$ become ^69^:

8 $${\Omega }_{n}^{z} \left( k \right) = - Im \left[ {\partial_{{k_{x} }} u_{nk} \left| { \partial_{{k_{x} }} u_{nk} - \partial_{{k_{y} }} u_{nk} } \right| \partial_{{k_{x} }} u_{nk} } \right]$$

in this equation, ${u}_{nk}$ is the periodic part of Bloch state *|nk* > *.* The Kubo-like formula is used to calculate Berry curvature as:

9 $${\Omega }_{n}^{z} \left( k \right) = - \mathop \sum \limits_{{}} \left( {f_{n} \left( k \right) - f_{m} \left( k \right)} \right)\frac{{Im\left[ {v_{nm,x} \left( k \right)v_{nm,y} \left( k \right)} \right]}}{{\left[ {\epsilon_{m} \left( k \right) - \epsilon_{n} \left( k \right) } \right]^{2} }}$$

Where $v_{nm,x} \left( k \right) = \psi_{mk} \left| {\hat{v}_{\alpha } } \right|\psi_{nk} = \frac{1}{\hbar } u_{mk} \left| {\partial_{{k_{\alpha } }} \widehat{H }\left( k \right)} \right|u_{nk}$ is a complex velocity, and $\epsilon_{m} \left( k \right)$ and $\epsilon_{n} \left( k \right)$ are both empty or filled up. Berry curvature has opposite signs in the VB and CB valleys. Now, the roughly evaluation of valley Hall conductivity for electrons for zero Kelvin near K valley become ^70^:

10 $$\sigma_{v}^{e} = \mathop \int \limits_{{\frac{{E_{g} }}{2}}}^{\infty } dE g\left( E \right) f\left( E \right)\Omega_{c,k} \approx \frac{{e^{2} }}{\hbar } \mathop \int \limits_{{\frac{{E_{g} }}{2}}}^{\infty } dE \frac{2}{{\pi \left( {\hbar v_{f} } \right)^{2} }} E \frac{{2b^{2} t^{2} }}{{E_{g}^{\prime 2} }} = \frac{{2b^{2} t^{2} }}{{\pi \hbar^{2 } v_{f}^{2} E^{\prime 2} }} \left( {E_{F}^{2} - \frac{{E_{g}^{2} }}{4}} \right) \frac{{e^{2} }}{\hbar }$$

here, g(E) = $\frac{2}{{\pi \hbar^{2 } v_{f}^{2} }}E$ is the electron density of states (DOS) at K point, and Fermi–Dirac at zero kelvin becomes delta function.

## Results and discussions

Monolayer TMDs as a direct gap semiconductor emerge advanced optical materials for device applications. Hsu et al.^71^ reported that strain modifies the wavefunctions, band curvature and optical matrix, which affects the binding energy of the K-K direct exciton and radiative lifetime. In this study, we determine the direct/indirect gap properties for TMD nanoribbons without/with strain fields, which play as an effective perturbation for modulating electronic properties. We have first studied the electronic structures of six compound of TMDs nanoribbons with applied X-tensile and Y-arc strain by the TB approach. Taking the MX_2_ nanoribbons as an example, the model of strained structure is shown in Fig. 1.

### Electronic properties

**Figure 2:**
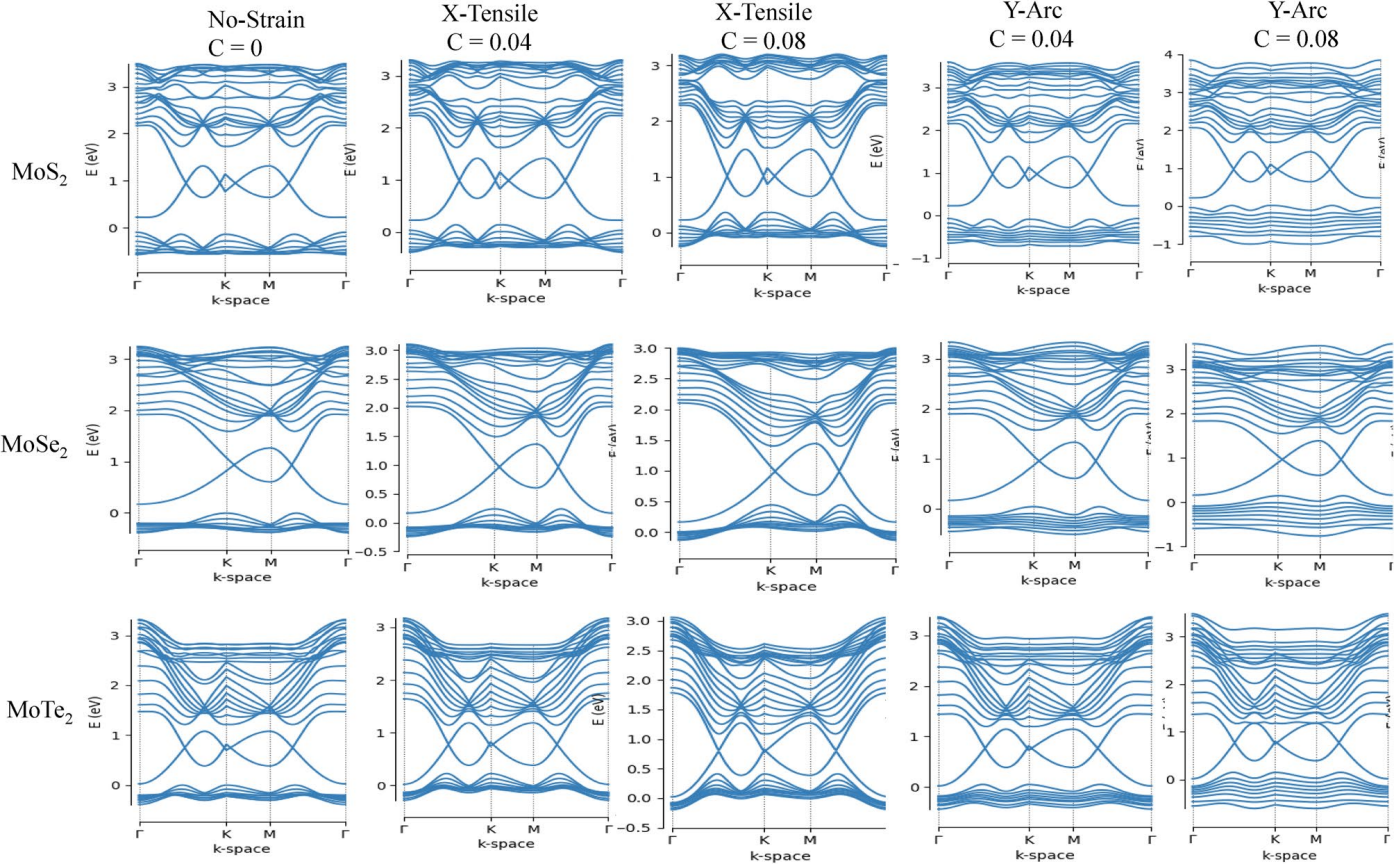
Electronic band structure of MoX_2_ (X = S, Se, Te) for two types of strain labeled as uniaxial X-tensile and Y-arc strain with c =  = 0, 0.04, 0.08.

**Figure 3:**
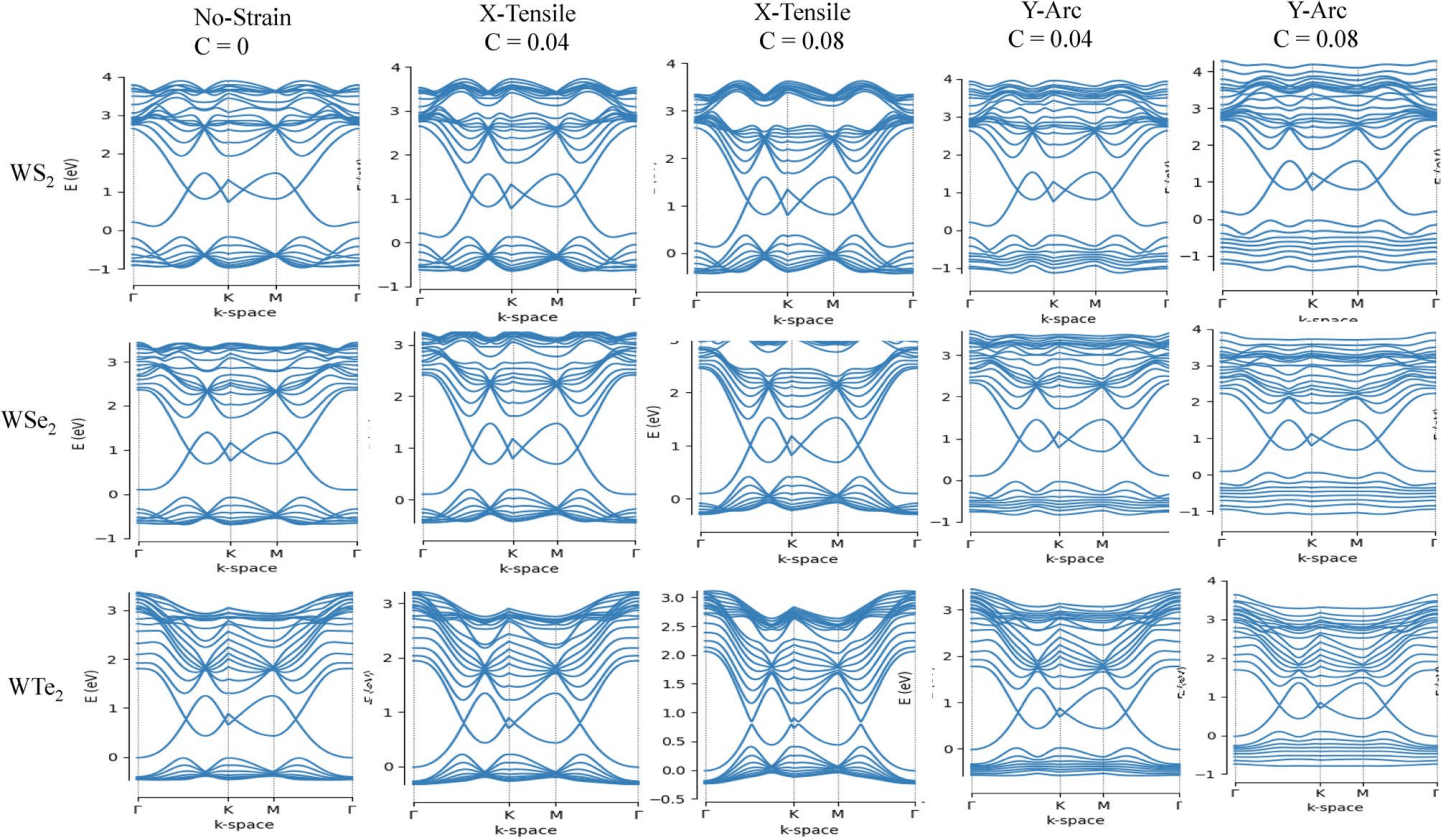
Electronic band structure of WX_2_ (X = S, Se, Te) for two types of strain labeled as uniaxial X-tensile and Y-arc strain with c = 0, 0.04, 0.08.

**Figure 4:**
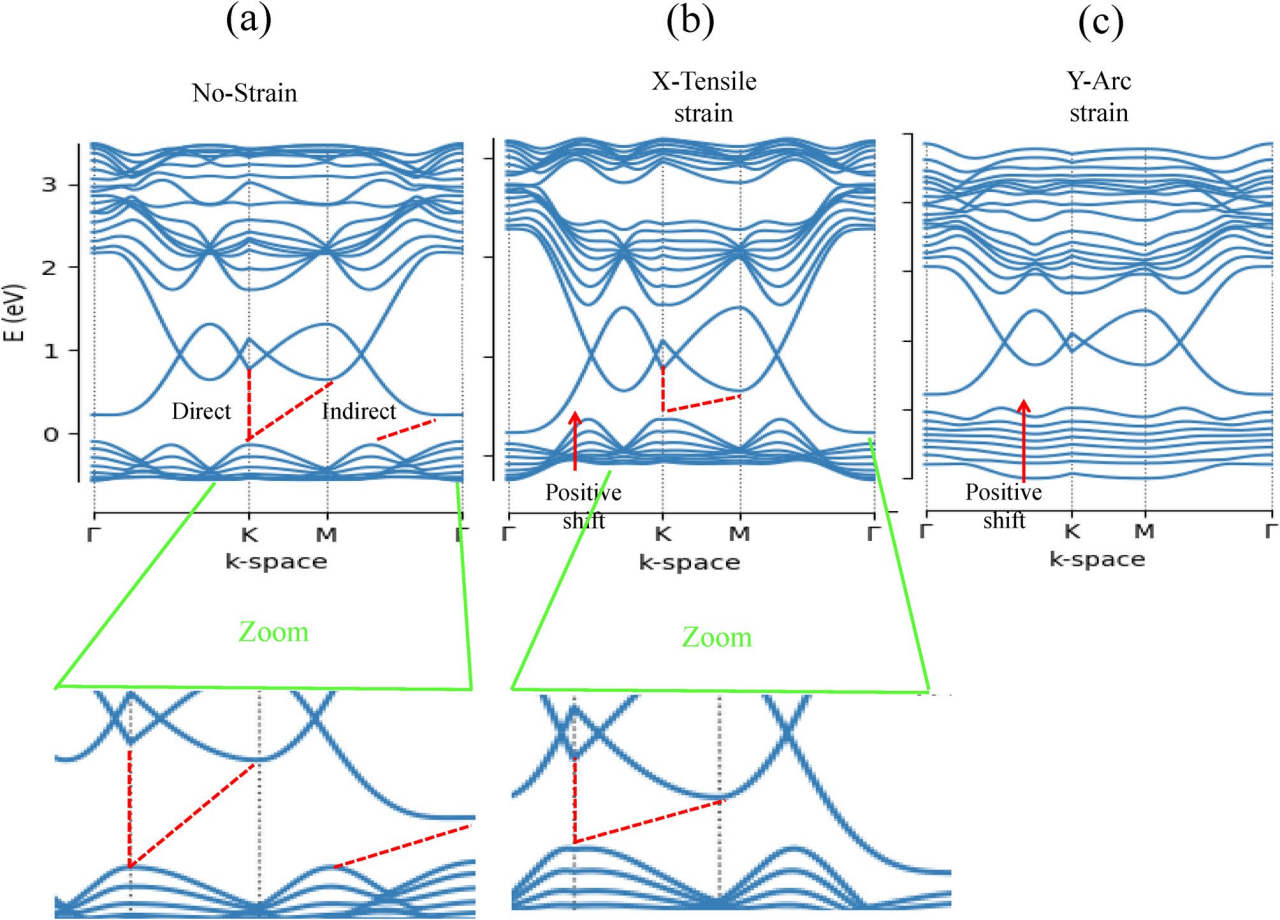
Electronic band structure of MoS_2_ (**a**) without strain and (**b**), (**c**) with two types of strain labeled as uniaxial X-tensile and Y-arc strain. Red dashed lines show the direct and indirect band gap, while red arrows represent shifted valance valleys towards positive energies after external stimuli. Bottom panels are zoom of exterma of valance and conduction bands.

The lattice deformation gauge fields by strain in TMD nanoribbons indicates a gap difference between two valley points, which can be source of valley Hall current in the strained nanoribbons. We perform TB approach to investigate the electronic properties of TMDs, i.e. MX_2_ nanoribbons. The calculated electronic band structure of six TMDs nanoribbons is shown in Figs. 2 and 3, indicating semiconductor behavior of TMD nanoribbons with indirect band gap except for MoTe_2_, where their band gap variations is presented in Tables 2 and 3. The nature of the band gap remains indirect for both type of strain, i.e. X-tensile and Y-arc strain ranging of 0.02, 0.04, 0.06, 0.08, 0.10, 0.15 and 0.2 (see supplementary information). The size of the band gap goes to zero through a X-tensile strain after 0.02, while for Y-arc strain decreases monotonically under strain of 0.6–0.8%. A valence band in all six compounds of Y-arc strained TMD nanoribbons is split and flatted, which is further enhanced by increasing strain and leads to decreasing the electrons mobility. Evaluation of electronic properties of TMD nanoribbons by considering the DFT calculations has been established in several reports^72,73^. The band gap values calculated from conventional DFT functional underestimate those values derived from the experimental results^74^. Various factors on the experimental side such as doping^75^ and the dielectric screening of the substrate^76^ affect band structure, which complicate the comparisons to theoretical band structure^74^. Huang et al. in their paper^77^ by using first-principles DFT calculations found that the dynamic energy barrier have been decreased by applying the stress and strain, where the phase transition of MoTe_2_ from 2H to 1 T’ controlled by biaxial or uniaxial tensile strain^77^. The electronic properties of TMD nanoribbons (Mo-,W-,S_2_,Se_2_) by implementing first-principles DFT calculations studied by Davelou et al.^78^. They showed that TMD nanoribbons with zigzag edges are always metallic regardless of the composition, the width or the edge structures, which is in good agreement with our results^78^. Furthermore, the effect of strain on the electronic and magnetic properties of the MoS_2_ nanoribbons has been investigated by first-principle calculation^79^. Where, the stretchable MoS_2_ is nonmagnetic and its band gap decreases with increasing strain, and direct band gap at weak strain changes to indirect band gap with increased strain up to 10%^79^, which are in good agreement with our results. The band gap response of MoTe_2_ for both type of strain is the same, where it remains metallic. The CB minimum and the VB maximum of strained MoTe_2_ are contributed and dominated from the d orbitals of Mo atoms and p orbitals of Te atoms^77,80^, which make the strain effect on band gap unambiguous. Figure 4 indicates the Electronic band structure of MoS_2_ without strain and with two types of strain, where valance valleys are shifted towards positive energies by red arrows after external stimuli. Red dashed lines in this figure show the direct and indirect band gap.

**Table 2:** Band gap variation of X-Tensile strain.

Strain/Band gap (eV)	0	0.02	0.04	0.06	0.08	0.1	0.15	0.2
MoS_2_	0.37	0.22	0	0	0	0	0	0
MoSe_2_	0.16	0	0	0	0	0	0	0
MoTe_2_	0	0	0	0	0	0	0	0
WS_2_	0.26	0.24	0	0	0	0	0	0
WSe_2_	0.17	0	0	0	0	0	0	0
WTe_2_	0.03	0	0	0	0	0	0	0

**Table 3:** Band gap variation of Y-arc strain.

Strain/Band gap (eV)	0	0.02	0.04	0.06	0.08	0.1	0.15	0.2
MoS_2_	0.37	0.35	0.31	0.25	0.2	0	0	0
MoSe_2_	0.16	0.15	0	0	0	0	0	0
MoTe_2_	0	0	0	0	0	0	0	0
WS_2_ (direct)	0.26	0.24	0.21	0.19	0.16	0	0	0
WSe_2_	0.17	0.15	0.14	0.11	0	0	0	0
WTe_2_	0.03	0.02	0.01	0	0	0	0	0

**Figure 5:**
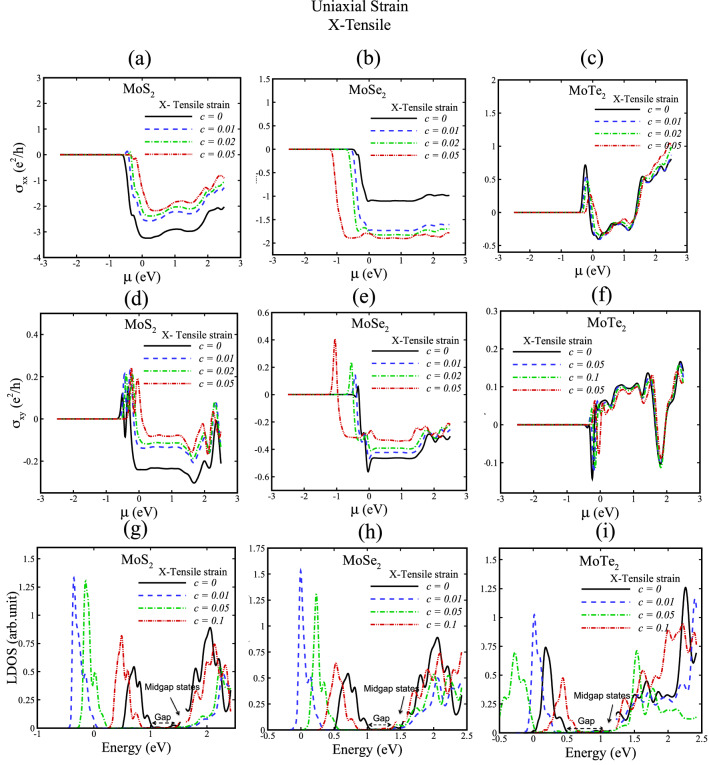
Hall conductivity and local density of states (LDOS) of MoX_2_ (X = S, Se, Te) for uniaxial X-tensile strain with c = 0, 0.01, 0.02, 0.05, 0.1. In (**g**), (**h**) and (**i**) gap and midgap states are indicated.

**Figure 6:**
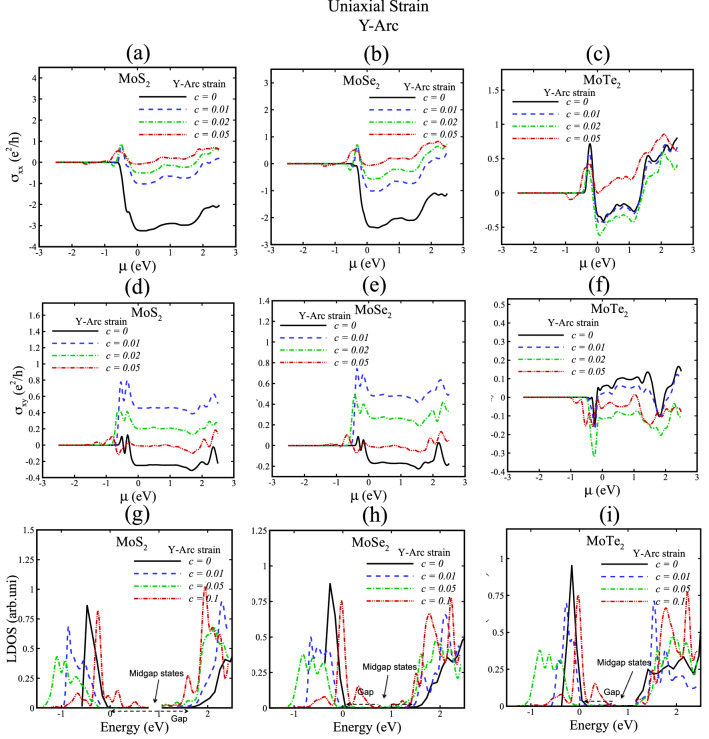
Hall conductivity and local density of states (LDOS) of MoX_2_ (X = S, Se, Te) for uniaxial Y-arc strain with c = 0, 0.01, 0.02, 0.05, 0.1. In (**g**), (**h**) and **(i**) gap and midgap states are indicated.

**Figure 7:**
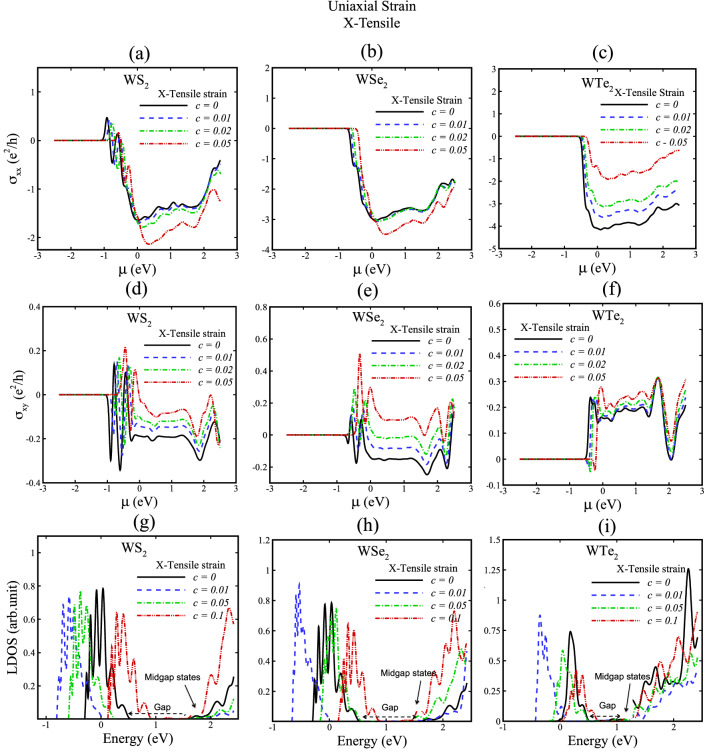
Hall conductivity and local density of states (LDOS) of WX_2_ (X = S, Se, Te) for uniaxial X-tensile strain with c = 0, 0.01, 0.02, 0.05, 0.1. In (**g**), (**h**) and (**i**) gap and midgap states are indicated.

**Figure 8:**
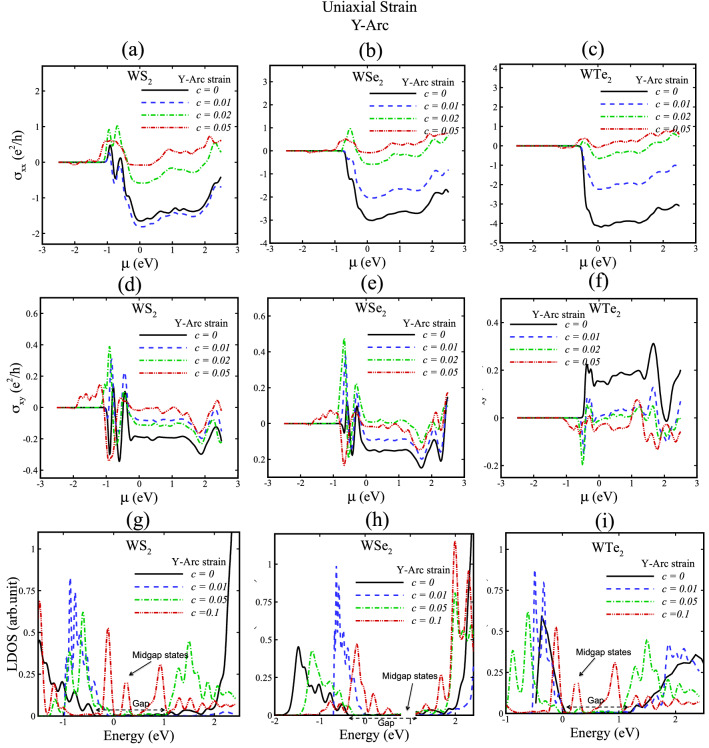
Hall conductivity and local density of states (LDOS) of WX_2_ (X = S, Se, Te) for uniaxial Y-arc strain with c = 0, 0.01, 0.02, 0.05, 0.1. In (**g**), (**h**) and (**i**) gap and midgap states are indicated.

In several reports have been revealed that the electronic and transport properties of TMD semiconductors can be crucially impacted by midgap states induced by dopants, defects, electric field and strain field, which can be native or intentionally incorporated in the crystal lattice^81–83^. The midgap states in the band structures of strained TMD nanoribbons in this study originate mainly from strain field, and tune with strain values. Herein, we calculated local density of states (LDOS) (commensurate with experimental STM data) to characterize the midgap states as shown in Figs. 5, 6, 7, 8g–i, and we have indicated the position of midgap states in these figures to avoid any confusion.

Herein, we apply the Slater-Koster TB-Hamiltonian to illustrate the strain effects on the electronic band structure (Eqs. 1, 2, 3) and the symmetry properties at specific k points relevant to the low-energy degrees of freedom, where modified hopping term is responsible for existence of midgap states. It’s worth to note that an alternative way to include the coupling terms such as strain and electromagnetic fields to TB-Hamiltonian has been established based on the effective k.p Hamiltonian, which expands the full TB model as (H_TB_(k) = H^0^_TB_(k) + H_strain_)^84^, where both methods take into account the midgap states as crucial impact on the electron transport properties of strained TMD nanoribbons.

### Valley hall conductivity

Intriguing properties in quantum materials are contributed by Berry curvature; such as intrinsic anomalous Hall effect. Exploring the evolution of Berry curvature due to external stimulus could be lead to emergent quantum transport properties. As mentioned in method section, Berry curvature (Eqs. 8, 9) is sensitive to changes of wavefunction and electronic band structure, which is tuned by external stimulus. For instance, in gated monolayer TMDs, controllable of VHE has been proposed by Rashba type spin orbit coupling, which needs strong displacement fields of 0.3–0.4 eV/A° in ionic liquid gated device^85–87^. We further determine that strain field can manipulate Berry curvature, where plays a significant role in exotic electronic states of quantum materials, such as the VHE. Herein, we apply two uniaxial strains to TMD nanoribbons for tuning of Berry curvature and investigate the changes of magnitude and sign of their valley Hall conductivity due to modified electronic structures.

Our calculation of band structure of strained TMD nanoribbons reveals that valence bands are shifted to positive energies in the K-space. Therefore, strain alters the sign of valence bands and Berry curvature, which leads to sign and magnitude change of valley Hall conductivity as shown in Figs. The valley-carried orbital magnetic moment characterize the valley degree experimentally^7,88,89^. In our work, the orbital magnetic moment for conduction and valance band in the presence of the strain fields is defined as: $m_{\nu z}^{{k\left( {k^{\prime } } \right)}} = - \nu \frac{{ed \left( {d^{\prime } } \right)}}{\hbar } \Omega_{{\nu k\left( {k^{\prime } } \right)z}} \left( k \right)$^90^; where Ω is the Berry curvature and ν =  ± stands for the valence and conduction band. This formula indicates that the magnitude and sign of magnetic moment depends on Berry curvature, which is tuned by strain field. Our findings reveal that strain field changes the order of d orbital energies of transition metal in TMD nanoribbons, which induces a crystal field splitting and lead to change of sign and magnitude of the valley Hall conductivity.

We now discuss how strained TMDs affect the valley Hall conductivities due to Berry curvature features. We calculate the transverse (σ_xx_) and valley Hall conductivity (σ_xy_) of TMD nanoribbons without strain and with X-tensile strain as represented in Figs. 5, 7, and for Y-arc strain in Figs. 6, 8. A theoretical hypothesis of VHE can be done based on Eqs. 7, 10. By varying the strain parameters; the magnitude and sign of the valley Hall conductivity can be tuned, where σ_xx_ and σ_xy_ in Figs. 5, 6, 7, 8 figure out this variations. Moreover, the observed small resistance of strained TMD nanoribbons in the absence of external magnetic field reveals the existence of induced pseudomagnetic potential in strained structures due to strain field.

### Pseudoelectric and pseudomagnetic field (Landau level)

Inhomogeneous strains in graphene can induce pseudomagnetic fields very similarly to real fields^93^. The induced magnetic field introduce multiple singularities in density of states as Landau level, which is separated by band gaps^91^. A strain gradient creates pseudoelectric and pseudomagnetic fields at strained structure, where high or low density of atoms and, hence, electrons (inhomogeneous charge distribution) are emerged as shown in marked regions in Fig. 1, which results in an pseudoelectric field. In Y-arc strained TMD nanoribbons, stretching of bonds cause the momentum K and K′ points of Dirac cones shift as δk from their unstrained positions in the reciprocal space. This δk as a momentum shift generates a pseudovector potential term eA/c^92^, which creates opposite signs of pseudomagnetic fields at the two valleys. The Y-arc strain creates rare and dense regions in the TMD nanoribbons, acting as two different materials in a superlattice. Pseudomagnetic fields are ± ẑ field direction for pseudo spin up and down, where the valley polarized states are formed due to reversal of pseudospins.

Local density of states (LDOS) of X-tensile strained TMD nanoribbons in Figs. 5 and 7g, h, i suggest that new electron states of conduction bands are created by increasing strain fields and electron valance band states are shifted toward Fermi energy due to induced pseudofields in strained TMD nanoribbons. Furthermore, Figs. 6 and 8g, h, i indicate the LDOS for Y-arc strained TMD nanoribbons, where maximized LDOS is located in the bottom of arc shaped. LDOS of Y-arc TMD nanoribbons confirms the emergence of new quantized Landau level, due to pseudomagnetic potential of inhomogeneous strain profile of Y-arc strained TMD nanoribbons.

## Conclusion

In conclusion, by employing TB approach we have investigated the electronic, and valley Hall conductivity of six TMD nanoribbons such as MoS_2_, MoSe_2_, MoTe_2_, WSe_2_, WSe_2_ and WTe_2_ considering X-tensile and Y-arc strain up to 20%. The nature of electronic structure is indirect for both type of strained TMD nanoribbons and a transition from semiconductor to metallic is observed for almost all TMD nanoribbons by enhancing strain fields, where the valance valleys are shifted towards positive energies. Furthermore, we note that anomalous valley Hall conductivity (AVHC) of TMD nanoribbons in the sign and magnitude altered with strain. The deformed hexagonal structure of reciprocal lattice in Brillouin zone by strain field moves the Dirac cones at the K and K′ points (changed momentum K → K + δk) in opposite direction, which interpreted as a pseudo-vector potential. Meanwhile, pseudomagnetic field due to gradient strain in TMDs affects electron valleys in opposite direction for K and K′ valleys. Moreover, stretching the TMDs lattice as Y-arc strain changes the local electron density, which creates an in-plane electric field. Pseudomagnetic field in strained TMD nanoribbons affects the Berry curvature and emerges new quantized Landau level in align with nonmonotonic change of AVHC. Our findings demonstrate that there is a coupling between the strain field and the valley degree of freedom in TMD nanoribbons, which leads to a significant advance in valley-dependent electronics as well as fundamental condensed matter physics.

## Supplementary Information

Supplementary Information.

## Data Availability

The datasets generated during and/or analyzed during the current study are available from the corresponding author on reasonable request. Supporting information is available online.
