# Supplementary material for: Strain engineering of electronic properties and anomalous valley hall conductivity of transition metal dichalcogenide nanoribbons
Source: Sci Rep. 2022 Jul 4;12:11285. doi: 10.1038/s41598-022-13398-5 (PMC9253103; doi:10.1038/s41598-022-13398-5)
Supplement: Supplementary file 1 — Supplementary Information. [file 41598_2022_13398_MOESM1_ESM.pdf]

Supplementary Information

Strain Engineering of Electronic Properties and Anomalous Valley Hall Conductivity of Transition Metal Dichalcogenide Nanoribbons

Farzaneh Shayeganfar[1*]

[1]Department of Physics and Energy Engineering, Amirkabir University of Technology, Tehran, Iran

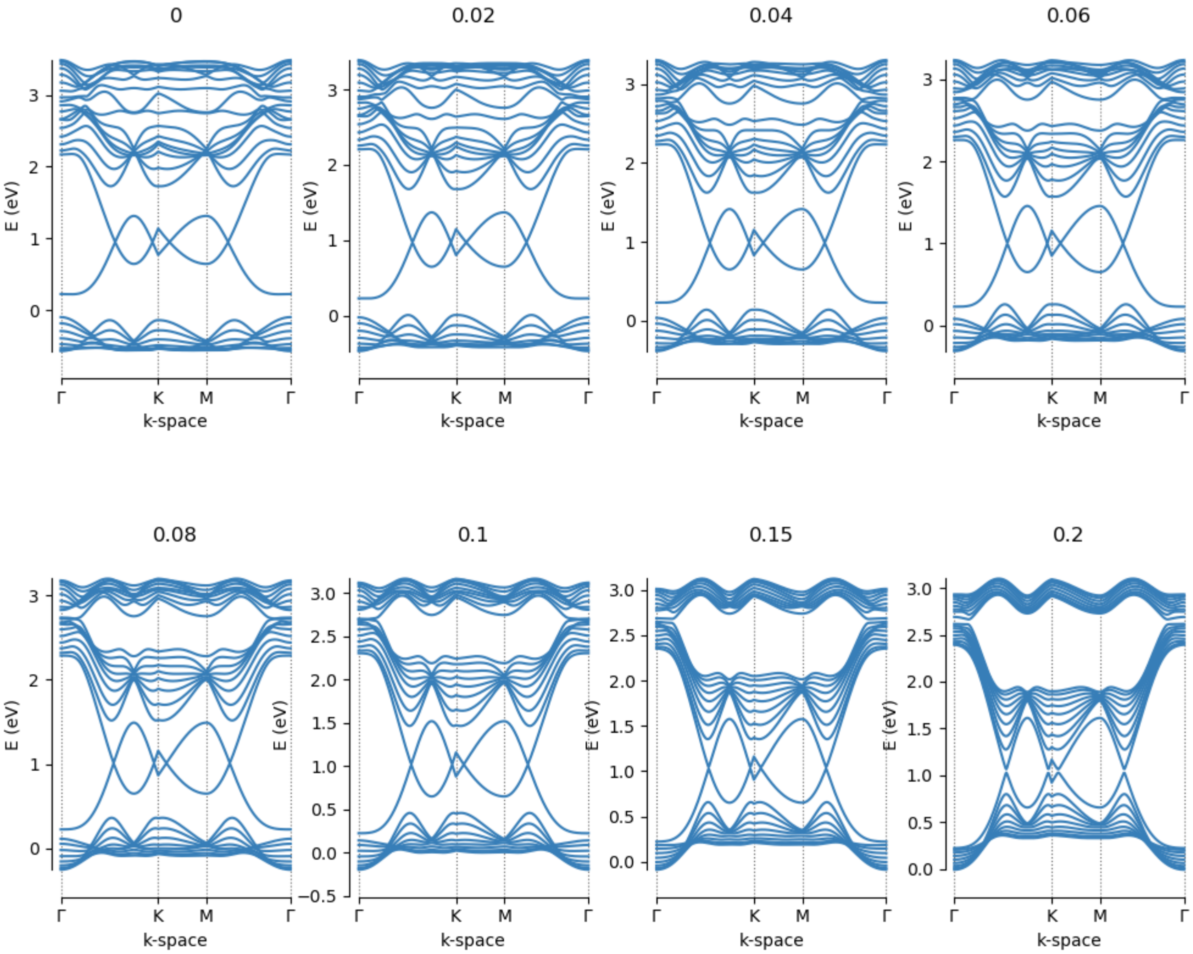

Fig S1. Electronic band structure of MoX_2 (X = S) for two types of strain labeled as uniaxial X-tensile strain with c = = 0, 0.02, 0.04, 0.06, 0.08, 0.1, 0.15, 0.2.

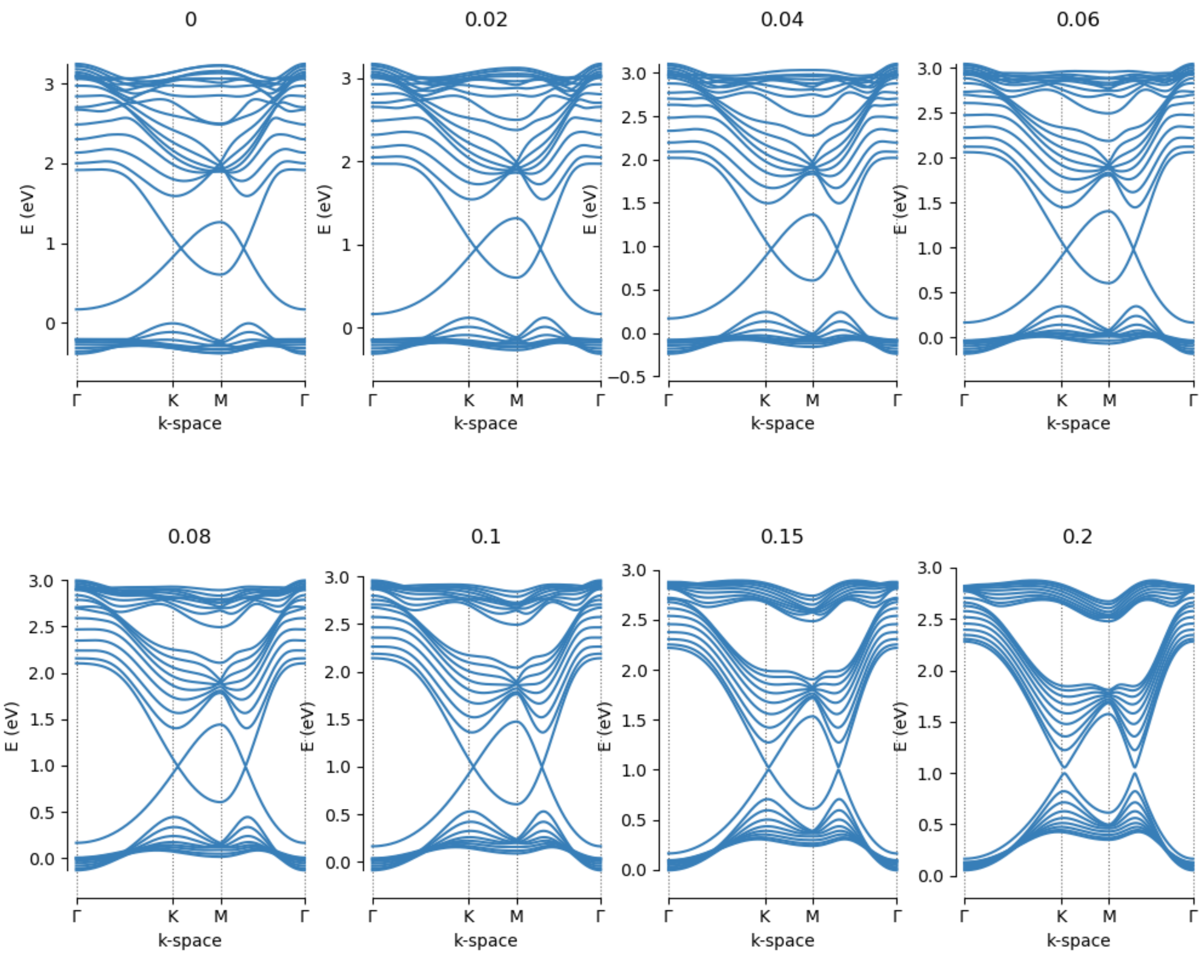

Figure S2. Electronic band structure of MoX_2 (X = Se) for two types of strain labeled as uniaxial X-tensile strain with c = 0, 0.02, 0.04, 0.06, 0.08, 0.1, 0.15, 0.2.

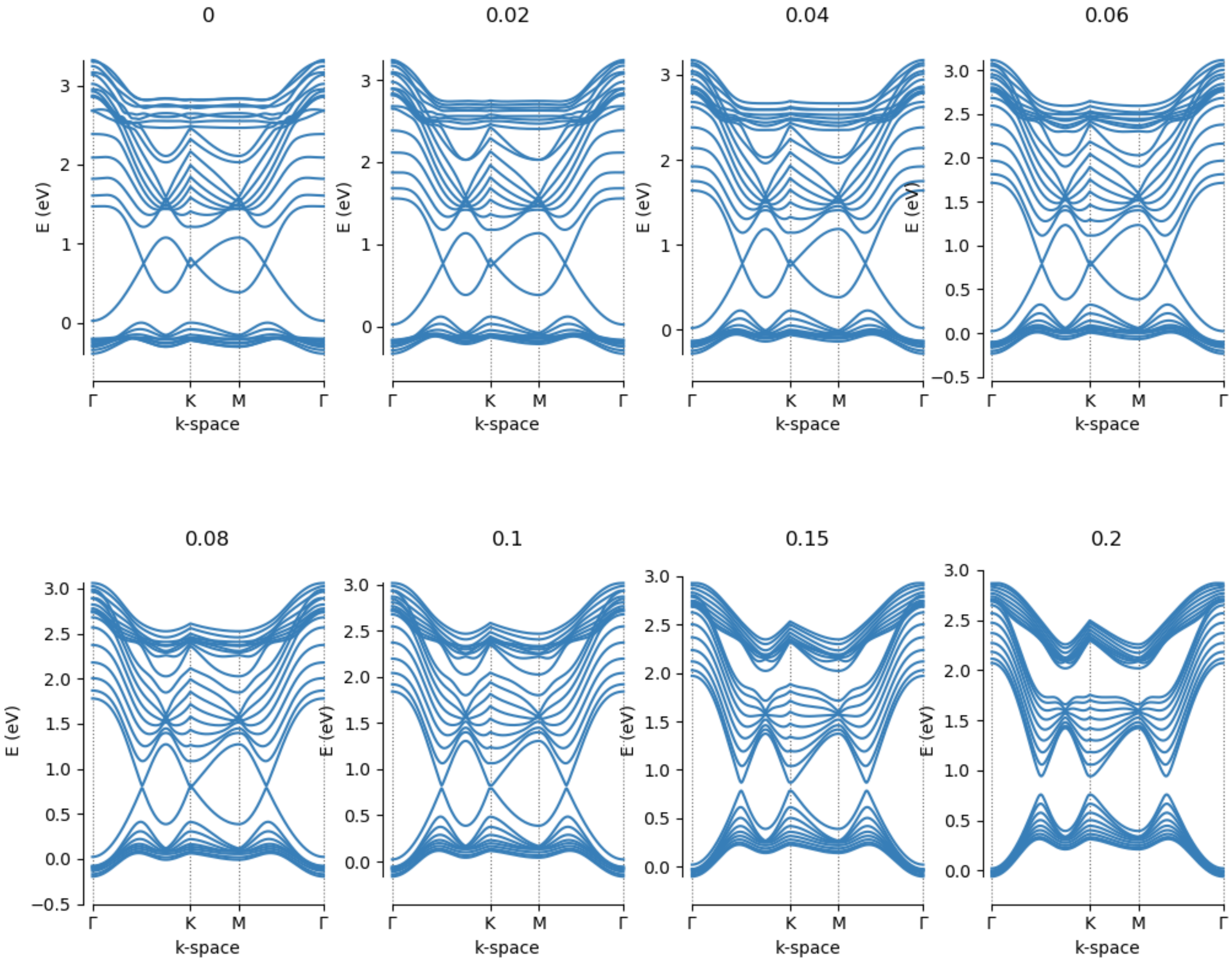

Figure S3. Electronic band structure of MoX_2 (X = Te) for two types of strain labeled as uniaxial X-tensile strain with c = = 0, 0.02, 0.04, 0.06, 0.08, 0.1, 0.15, 0.2.

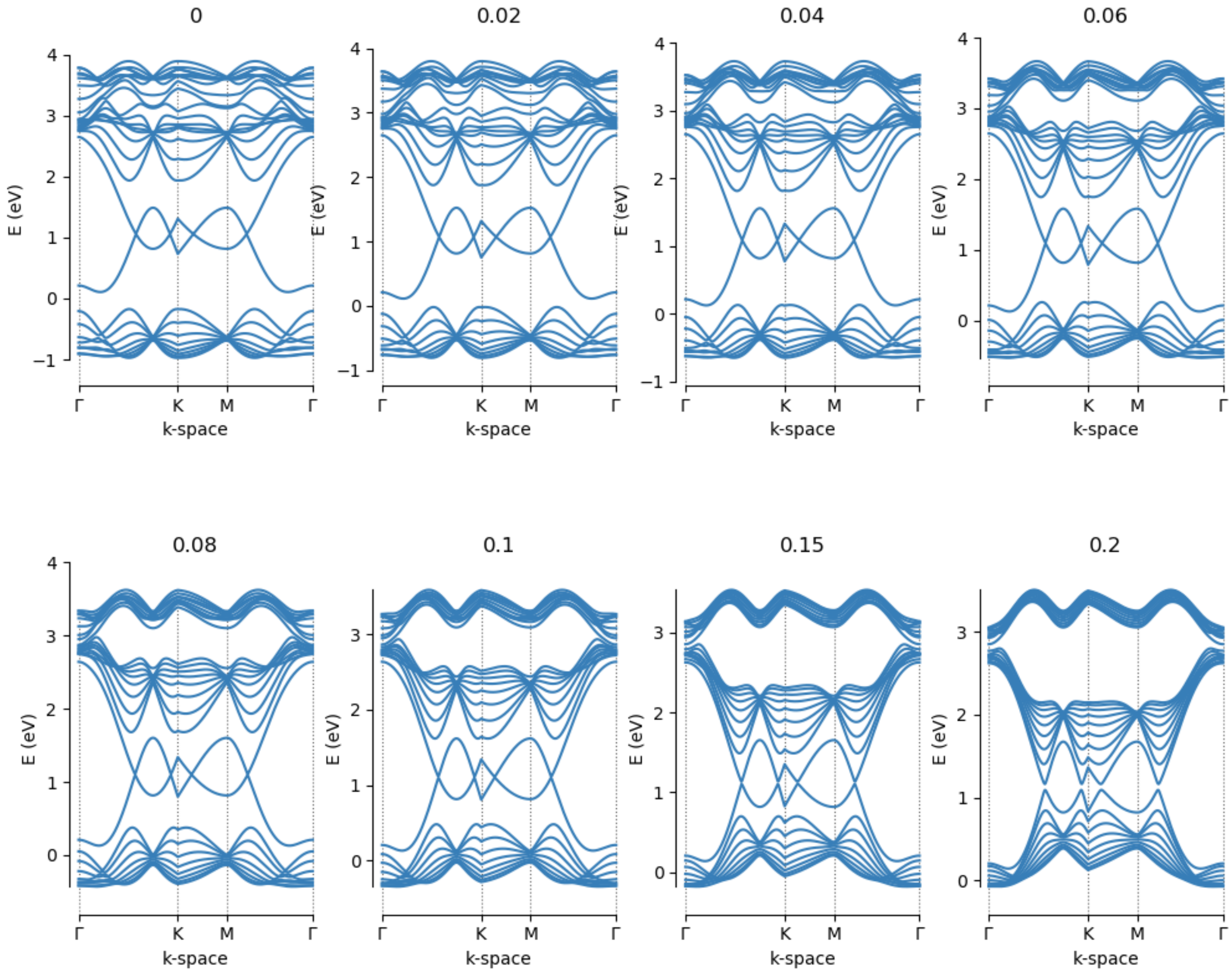

Figure S4. Electronic band structure of WX_2 (X = S) for two types of strain labeled as uniaxial X-tensile strain with c = = 0, 0.02, 0.04, 0.06, 0.08, 0.1, 0.15, 0.2.

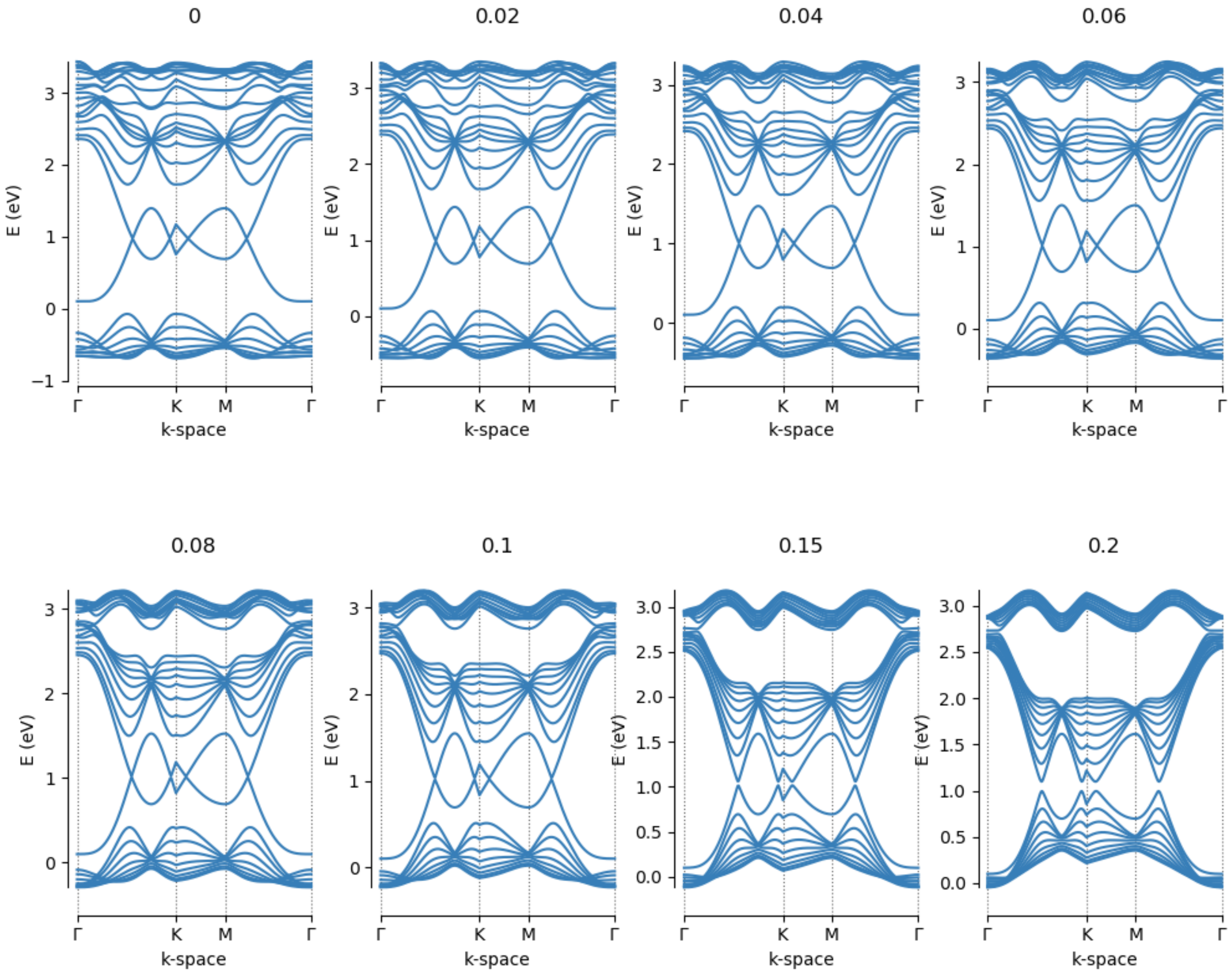

Figure S5. Electronic band structure of WX_2 (X = Se) for two types of strain labeled as uniaxial X-tensile strain with c = = 0, 0.02, 0.04, 0.06, 0.08, 0.1, 0.15, 0.2.

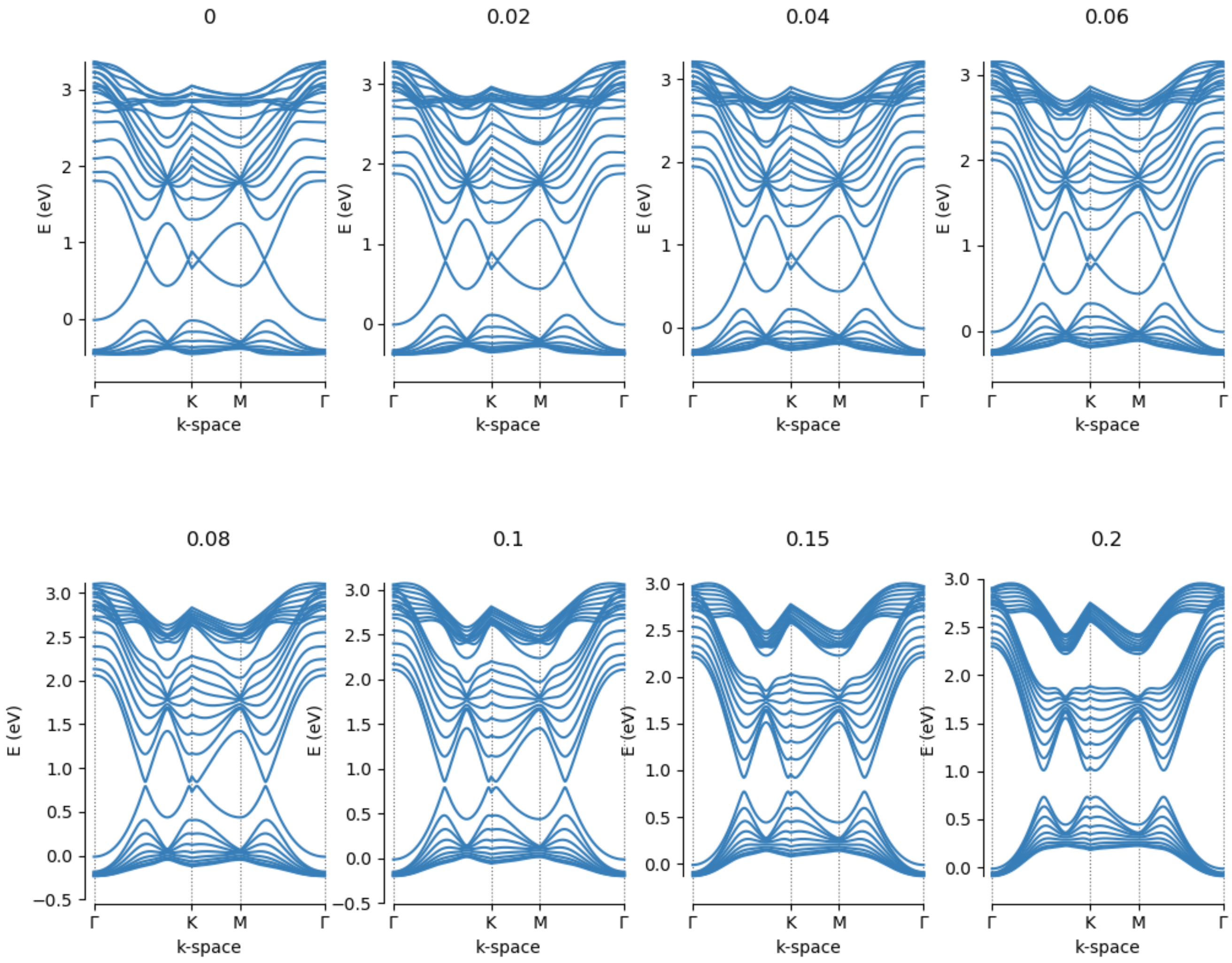

Figure S6. Electronic band structure of WX_2 (X = Te) for two types of strain labeled as uniaxial X-tensile strain with c = = 0, 0.02, 0.04, 0.06, 0.08, 0.1, 0.15, 0.2.

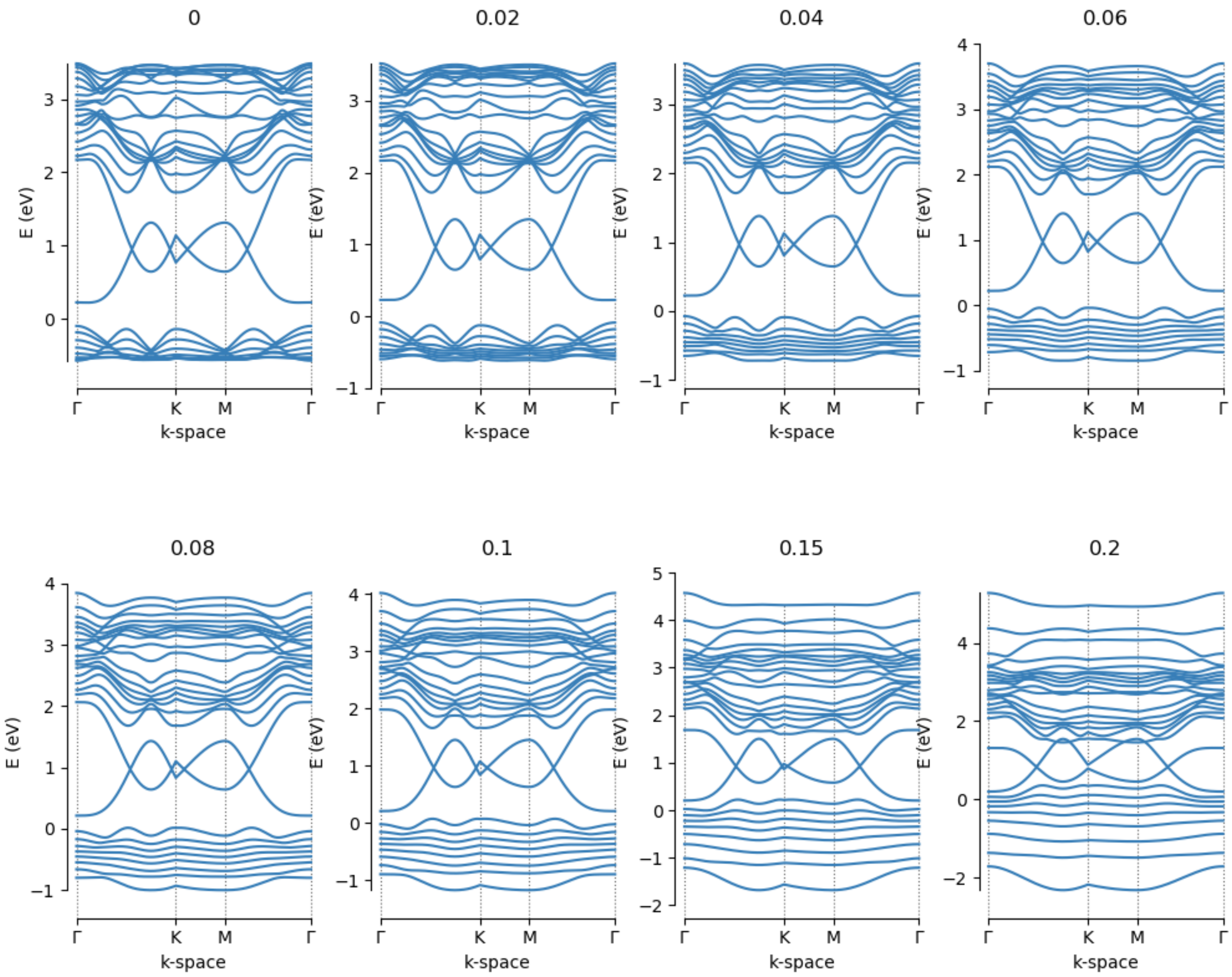

Figure S7. Electronic band structure of MoX_2 (X = S) for two types of strain labeled as uniaxial Y-arc strain with c = = 0, 0.02, 0.04, 0.06, 0.08, 0.1, 0.15, 0.2.

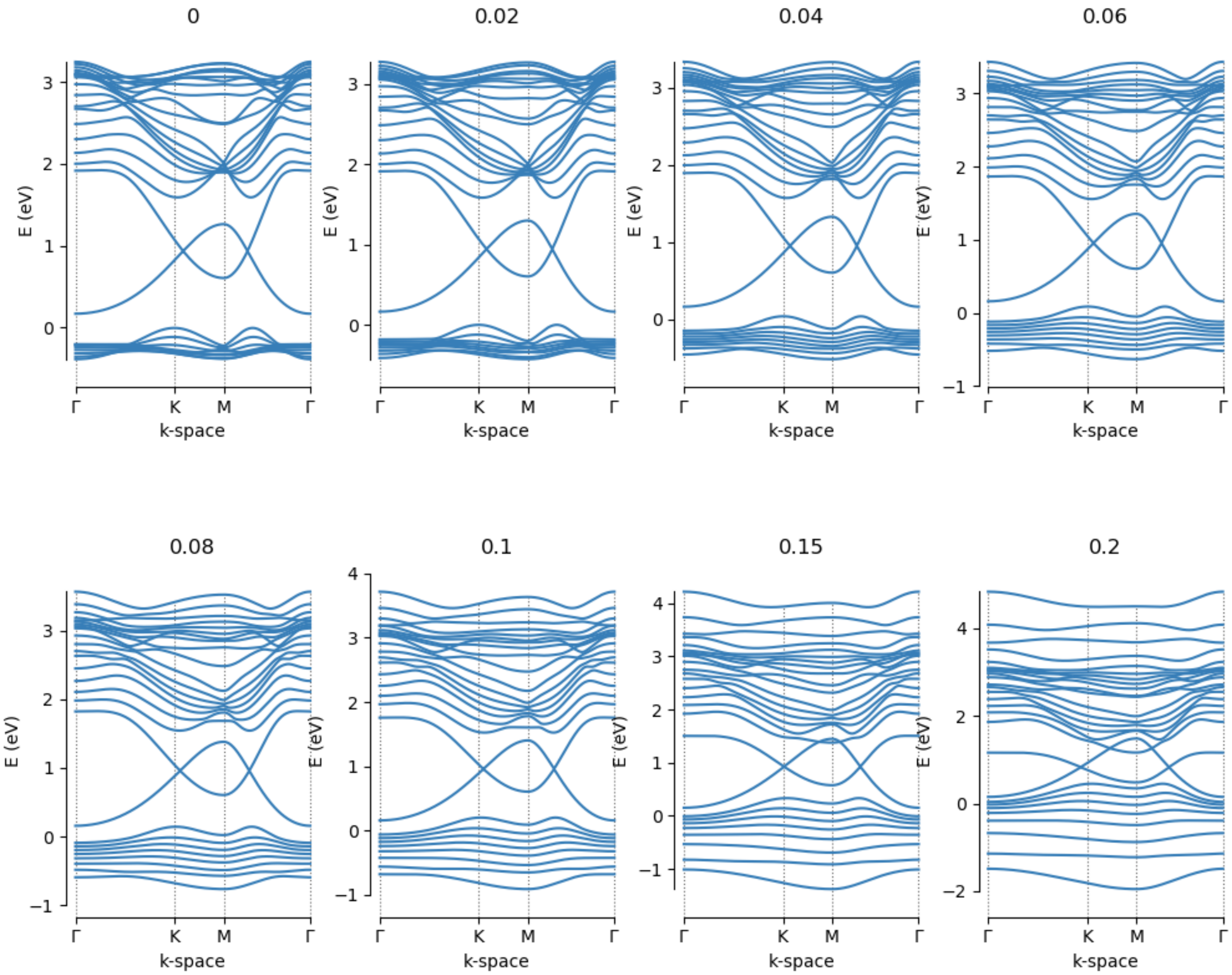

Figure S8. Electronic band structure of MoX_2 (X = Se) for two types of strain labeled as uniaxial Y-arc strain with c = = 0, 0.02, 0.04, 0.06, 0.08, 0.1, 0.15, 0.2.

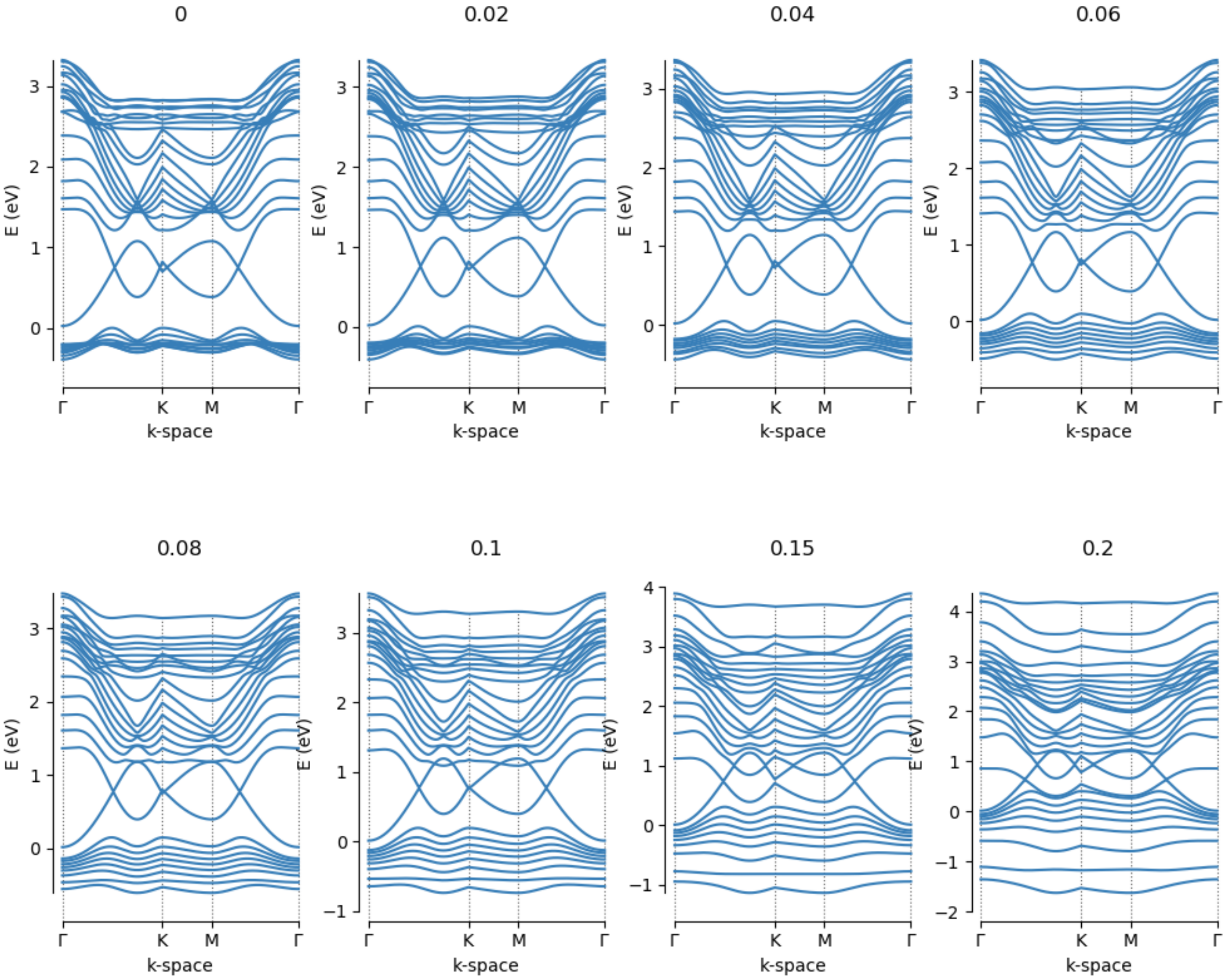

Figure S9. Electronic band structure of MoX_2 (X = Te) for two types of strain labeled as uniaxial Y-arc strain with c = = 0, 0.02, 0.04, 0.06, 0.08, 0.1, 0.15, 0.2.

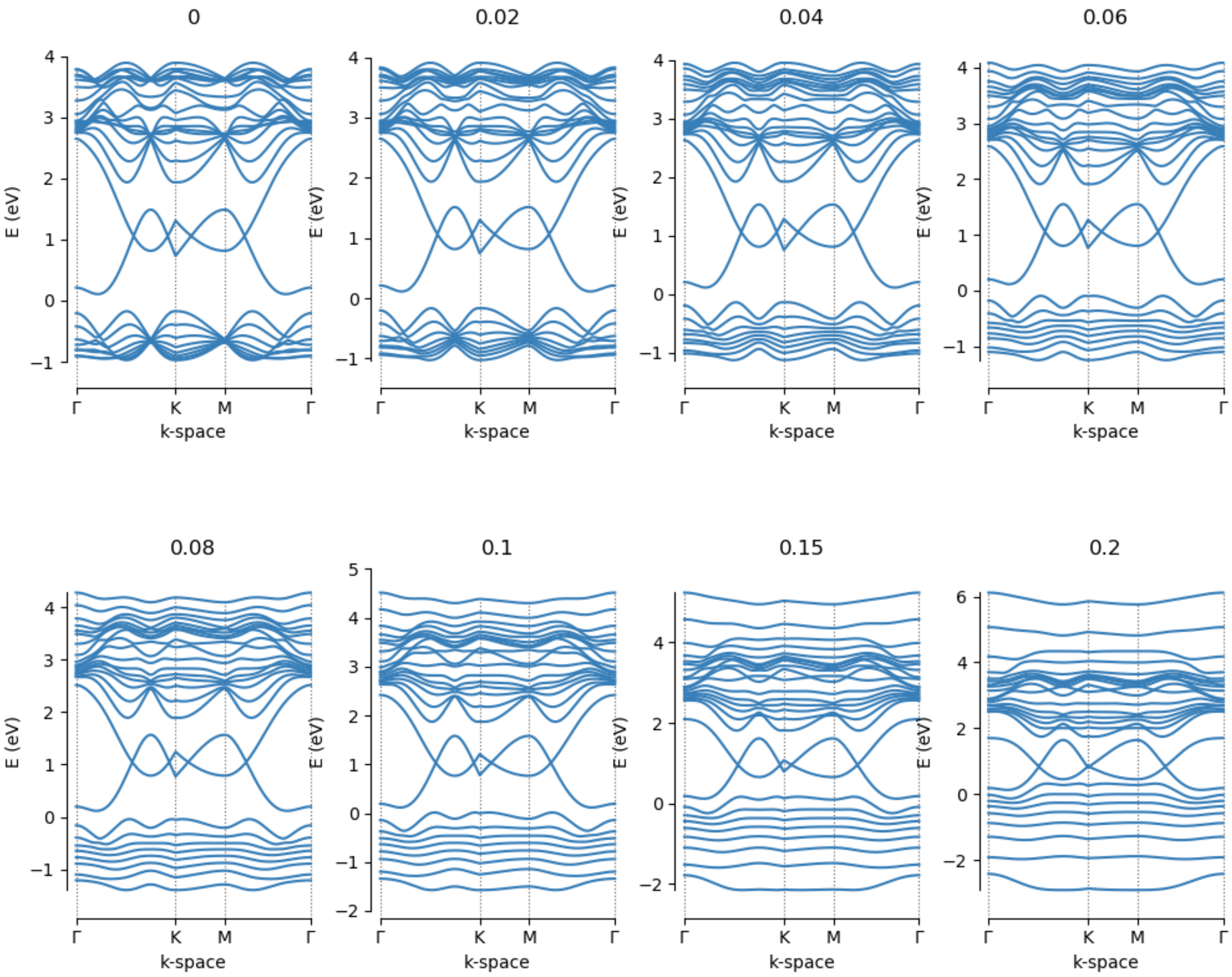

Figure S10. Electronic band structure of WX_2 (X = S) for two types of strain labeled as uniaxial Y-arc strain with c = = 0, 0.02, 0.04, 0.06, 0.08, 0.1, 0.15, 0.2.

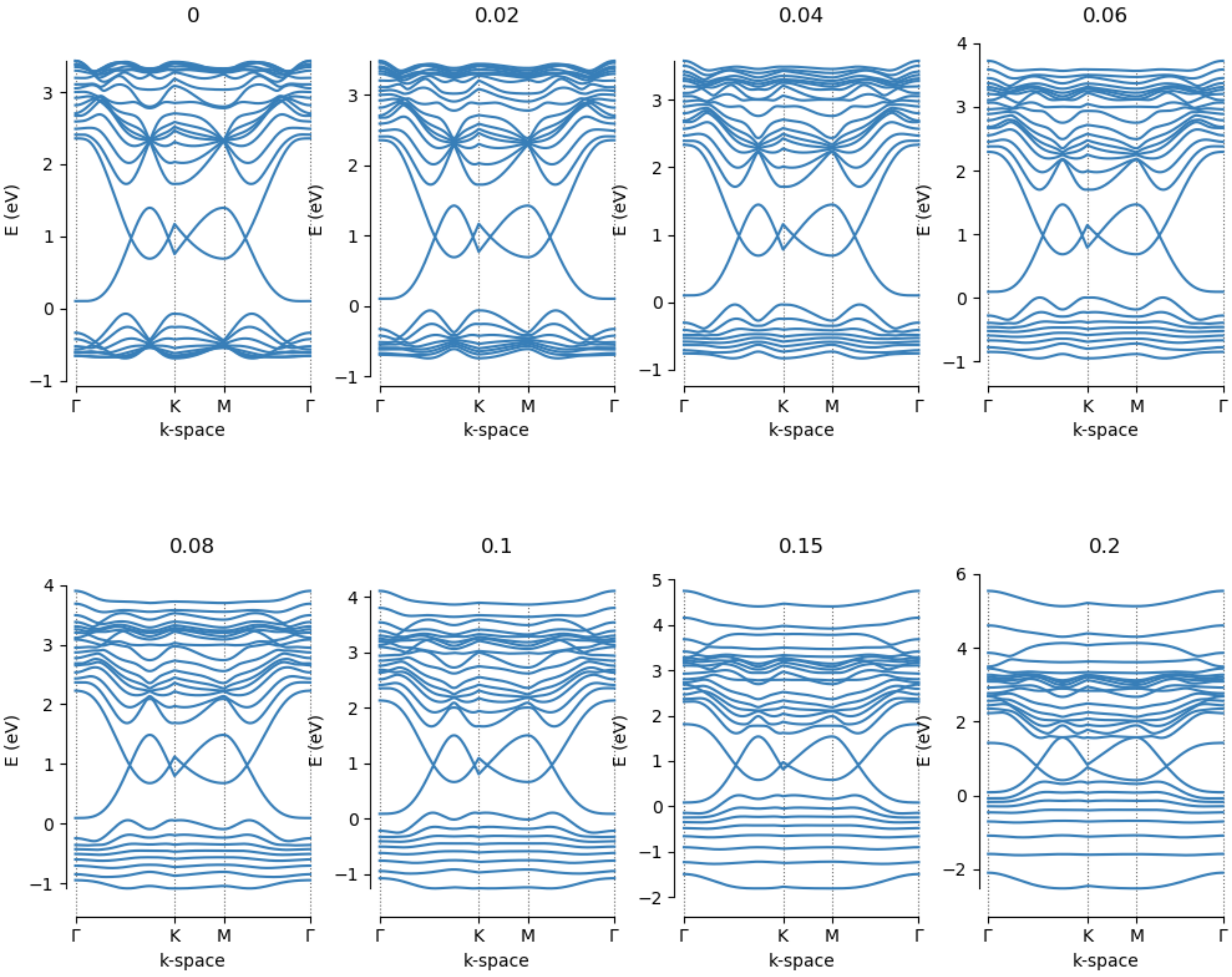

Figure S11. Electronic band structure of WX_2 (X = Se) for two types of strain labeled as uniaxial Y-arc strain with c = = 0, 0.02, 0.04, 0.06, 0.08, 0.1, 0.15, 0.2.

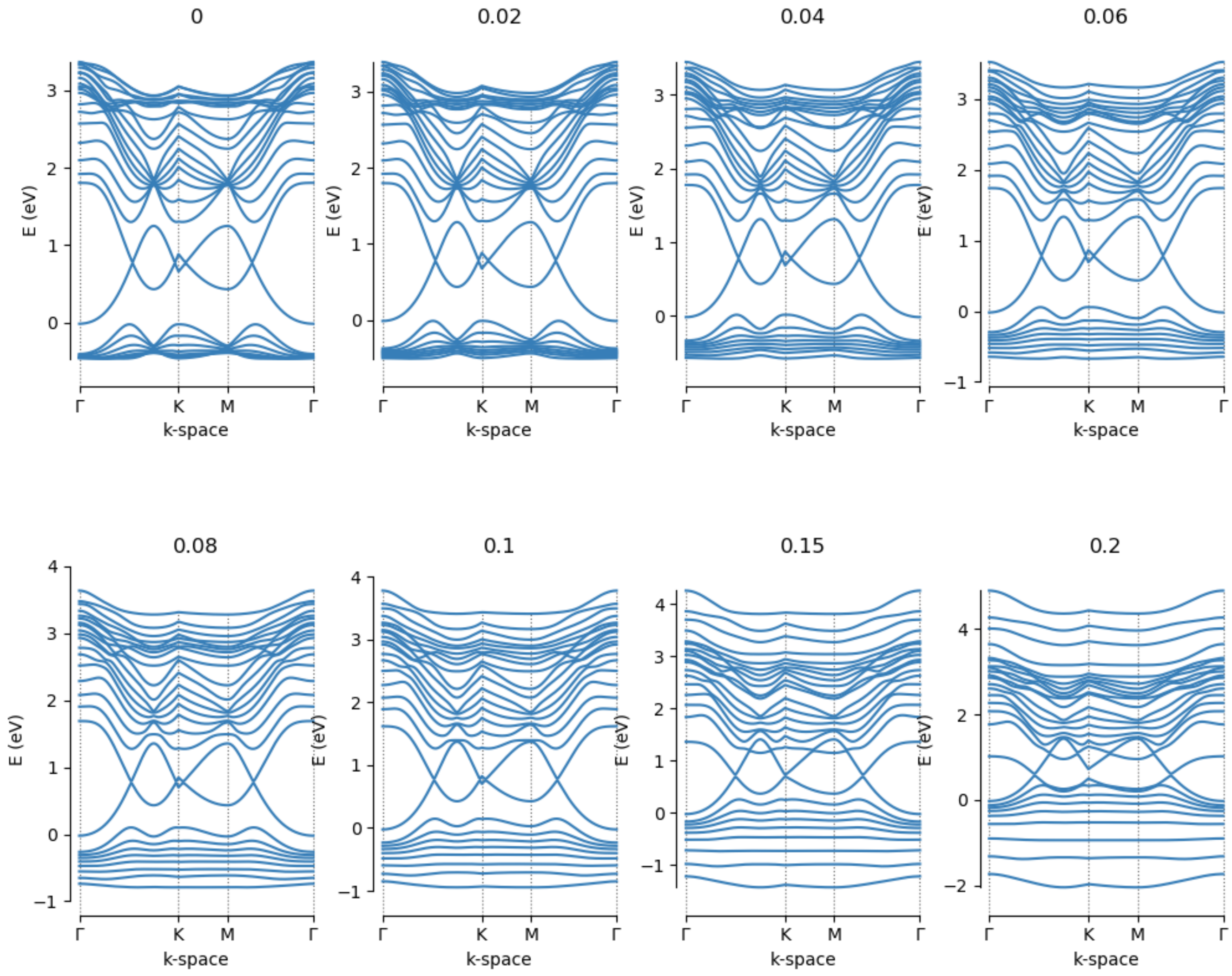

Figure S12. Electronic band structure of WX_2 (X = Se) for two types of strain labeled as uniaxial Y-arc strain with c = = 0, 0.02, 0.04, 0.06, 0.08, 0.1, 0.15, 0.2.
